# Microglia/Astrocytes–Glioblastoma Crosstalk: Crucial Molecular Mechanisms and Microenvironmental Factors

**DOI:** 10.3389/fncel.2018.00235

**Published:** 2018-08-03

**Authors:** Diana Matias, Joana Balça-Silva, Grazielle C. da Graça, Caroline M. Wanjiru, Lucy W. Macharia, Carla Pires Nascimento, Natalia R. Roque, Juliana M. Coelho-Aguiar, Cláudia M. Pereira, Marcos F. Dos Santos, Luciana S. Pessoa, Flavia R. S. Lima, Alberto Schanaider, Valéria P. Ferrer, Tania Cristina Leite de Sampaio e Spohr, Vivaldo Moura-Neto

**Affiliations:** ^1^Instituto Estadual do Cérebro Paulo Niemeyer – Secretaria de Estado de Saúde, Rio de Janeiro, Brazil; ^2^Instituto de Ciências Biomédicas, Universidade Federal do Rio de Janeiro, Rio de Janeiro, Brazil; ^3^Center for Neuroscience and Cell Biology and Institute for Biomedical Imaging and Life Sciences Consortium, University of Coimbra, Coimbra, Portugal; ^4^Faculty of Medicine, University of Coimbra, Coimbra, Portugal; ^5^Programa de Pós-Graduação em Anatomia Patológica, Faculdade de Medicina, Universidade Federal do Rio de Janeiro, Rio de Janeiro, Brazil; ^6^Universidade do Grande Rio (Unigranrio), Duque de Caxias, Brazil; ^7^Centro de Cirurgia Experimental do Departamento de Cirurgia da Faculdade de Medicina, Universidade Federal do Rio de Janeiro, Rio de Janeiro, Brazil

**Keywords:** glioblastoma, astrocytes, microglia, crosstalk, cytokines, molecular mechanisms, microenvironmental factors, communication mechanisms

## Abstract

In recent years, the functions of glial cells, namely, astrocytes and microglia, have gained prominence in several diseases of the central nervous system, especially in glioblastoma (GB), the most malignant primary brain tumor that leads to poor clinical outcomes. Studies showed that microglial cells or astrocytes play a critical role in promoting GB growth. Based on the recent findings, the complex network of the interaction between microglial/astrocytes cells and GB may constitute a potential therapeutic target to overcome tumor malignancy. In the present review, we summarize the most important mechanisms and functions of the molecular factors involved in the microglia or astrocytes–GB interactions, which is particularly the alterations that occur in the cell’s extracellular matrix and the cytoskeleton. We overview the cytokines, chemokines, neurotrophic, morphogenic, metabolic factors, and non-coding RNAs actions crucial to these interactions. We have also discussed the most recent studies regarding the mechanisms of transportation and communication between microglial/astrocytes – GB cells, namely through the ABC transporters or by extracellular vesicles. Lastly, we highlight the therapeutic challenges and improvements regarding the crosstalk between these glial cells and GB.

## Introduction

Glioblastoma (GB) is the most malignant primary tumor that affects the central nervous system (CNS) (Louis et al., 2016). Besides the conventional treatment to these tumors is complex, and based on surgery, radiotherapy, and chemotherapy with the *gold-standard* temozolomide (TMZ), the GB patient’s survival rate remains about 15 months after diagnosis (Stupp et al., 2007, 2009). In addition, the poor efficacy of TMZ has led the scientific community to find new therapeutic strategies that could be used for effective GB treatment using new substances or FDA-approved drugs against gliomas (Balça-Silva et al., 2015; Matias et al., 2017a). However, most of these substances do not have the ability to cross the blood–brain barrier (BBB), the biggest challenge to the passage of chemotherapeutics to the brain (Dubois et al., 2014). This barrier not only is composed mostly of endothelial cells, pericytes, fibroblasts, neurons, and basal membranes but also receives support from glial cells, such as astrocytes and microglia (Dubois et al., 2014; Zhao et al., 2017). During glioma progression, the BBB is compromised that allows the entrance of immune cells from blood, which in turn, promotes neuroinflammation. However, these alterations induce the chemoattraction and activation of glial cells. In fact, microglial cells produce high levels of proinflammatory molecules, such as nitric oxide (NO) and tumor necrosis factor alpha (TNF-α) which induce the BBB breakdown (Zhao et al., 2017). On the other hand, the tumor cells can induce the astrocytic activation by releasing interleukins (ILs), such as IL-1, and consequently disrupting the astrocyte–BBB junctions (Guan et al., 2018). Overall, these inflammatory alterations contribute to create an imbalance in the BBB function in the context of brain tumors like GB.

In fact, the mechanisms that support the GB’s resistance ability have been recently discussed, and it is already known that GB heterogeneity is a crucial reason to that resistance, due to communication between tumor and tumor parenchyma entities (Hambardzumyan et al., 2016).

Among various cells of the tumor microenvironment (TME), among the glial cells, like astrocytes and the microglial cells, are the most common cellular entities that interact with the GB and, consequently, contribute to their tumor growth (Gieryng et al., 2017b; Roos et al., 2017; Roesch et al., 2018).

Several studies using GB patient biopsies and *in vivo* animal models showed that the tumor mass is composed of 30–50% of glioma-associated microglia/macrophages (GAMs) (Roggendorf et al., 1996; Olah et al., 2012; Carvalho da Fonseca et al., 2014; Garcia et al., 2014; Zhang et al., 2015). Tumor cells have the ability to evade immune cells by creating an immunosuppressive microenvironment by releasing immunosuppressive factors, such as cytokines, chemokines, neurotrophic, and morphogenic factors, among others (Roggendorf et al., 1996; Olah et al., 2012; Garcia et al., 2014; Zhang et al., 2015; da Fonseca et al., 2016). In GBs, microglial cells have been shown to have a pro-tumor phenotype that is associated with the M2-like phenotype of macrophages due to its expression of specific factors, such as ILs, transforming growth factor beta 1 (TGF-β1), monocyte chemoattractant protein (MCP-1), and prostaglandin E2 (PGE-2) (Li and Graeber, 2012). On the other hand, GBs also induce alterations on astrocytes, turning them more reactive (Roessler et al., 1995; Guan et al., 2018). At the same time, the glial cells from TME also release factors that support the GB growth. Among those factors it has been previously demonstrated that CD11b+/Cd45-microglial cells are located around the tumor and express arginase-1 (ARG-1), which in turn stimulates the tumor proliferation (Zhang et al., 2015). Moreover, GB establishes direct contact with astrocytes/microglial cells through extracellular vesicles (EVs). EVs can transport important molecules, such as miRNAs and cytokines, that will turn astrocytes more reactive and, at the same time, induce M2-like phenotype on microglial cells (van der Vos et al., 2016; Oushy et al., 2018).

In this regard, the manipulation of microglia/astrocytes–GB crosstalk may be on the basis of many important and potential therapeutic strategies for the successful treatment of GB. Recently, it has been demonstrated that targeting microglial cells and astrocytes may be a potential tool for manipulating the GB growth (Ye et al., 2012; da Fonseca et al., 2016).

Taking this knowledge into account, many studies have recently focused on the identification of the principal targets and the most useful strategies to treat GB. In glioma-bearing mice, the blockage of CSF-1R, using the anti-CSF-1R antibody, known as Pexidartinib (PLX3397), is observed to significantly decrease tumor infiltration of GAMs leading to a reduction of the tumor volume (Pyonteck et al., 2013).

In the following sections, we will describe the physiological, biochemical, and microenvironmental functions of microglia/astrocytes with GB cells interaction, the molecular mechanisms underlying their crosstalk, their impact on tumor malignancy, and how this can be translated in a therapeutic purpose.

## Gliomas: a Deadly Threat

Among the glial cells, astrocytes and oligodendrocyte precursor cells (OPCs), which can differentiate into oligodendrocytes, are derived from stem-like radial glial cells in ventricular and subventricular zones during embryogenesis. Recently, one of the hypotheses to explain the origin of gliomas has been supported by several genetic and epigenetic mutations that occur on normal astrocytes, oligodendrocytes, and OPCs. Consequently, these mutations can give rise to gliomas. Among the most known are the oligodendrogliomas, oligoastrocytomas, or astrocytomas (Rycaj and Tang, 2015; Zong et al., 2015).

Gliomas are the most common primary malignant tumors of CNS in adults (Thakkar et al., 2014), 50% of which is GB. According to the World Health Organization’s (WHO) classification, they are ranked as the most malignant astrocytoma (Grade IV) (Ringertz, 1950; Paff et al., 2014; Urbańska et al., 2014; Louis et al., 2016) and will form the basis of this review.

Glioblastomas are mainly characterized by nuclear atypia, cellular pleomorphism, mitotic activity, diffuse growth pattern, microvascular proliferation, and/or necrosis, which constitute the main diagnostic features (Faria et al., 2006; Urbańska et al., 2014). These characteristics are the result of the genomic instability and the deregulation of several molecular signaling pathways that culminate in a high resistance to chemotherapy (Louis et al., 2016; Wirsching and Weller, 2016). The therapeutic approaches to GB treatment are highly aggressive, like surgery, radiotherapy, and chemotherapy with TMZ, the *gold-standard* treatment. However, only 5% of the patients survive >5 years (Ohgaki and Kleihues, 2007; Ostrom et al., 2014).

In this vein, the low survival of GB patients is due to the mechanisms of resistance and high heterogeneity (Stupp et al., 2007). This heterogeneity seems to be an indispensable key for tumor progression, resistance, metastasis, and tumor recurrence (Patel et al., 2014; Wang et al., 2017). Moreover, two types of tumor heterogeneity coexist: intrinsic and extrinsic. Currently, it is known that the intrinsic heterogeneity present in tumors is defined by the presence of cell niches with distinct phenotypic characteristics. It is believed by the scientific community that the presence of a subpopulation known as cancer stem-like cells (CSCs) is responsible for this heterogeneity. The maintenance of the different phenotypes also depends on the interaction with the microenvironment. These interactions are derived from the activation of several pathways through membrane receptors and their ligands, such as growth factors and cytokines as the epidermal growth factor receptor (EGFR) or IL-6, respectively. In addition, these factors are essential for the recruitment of parenchymal cells, such as endothelial cells, astrocytes, and microglia, that ultimately infiltrate to the tumor, helping tumor progression, which is referred to as extrinsic heterogeneity (Wang et al., 2009; Fonseca et al., 2012; Patel et al., 2014).

The conventional treatments contribute to the maintenance of GB heterogeneity. The main goals of surgery are to establish a pathological diagnosis, relieve mass effect, and achieve a gross total resection to facilitate adjuvant therapy (Moiyadi and Shetty, 2012). Removing of 98%, or even more, of the tumor mass identified in magnetic resonance imaging (MRI) conduct to a statistically significant increase in patient survival rate (Miller et al., 1988). Regarding radiotherapy, it is well known that it contributes to the proliferation of GSCs (Bao et al., 2006). In fact, the radiation has been associated to an increased number of non-tumor cells, such as reactive astrocytes and microglia/macrophages in the tumor bulk (Hwang et al., 2006; Tabatabaei et al., 2017). Recently, high levels of MCP-1, IL-8, and macrophage inflammatory protein-1 alpha (MIP-1a), which are associated with inflammatory response (Tabatabaei et al., 2017), were observed in irradiated GB tissues. Our group already showed that the TMZ does not significantly affect the glial cells proliferation, as well as microglial cells and astrocytes (Kahn et al., 2012). Overall, surgery, radiotherapy, and/or chemotherapy are still the conventional therapeutic arms against GB, although they are quite ineffective.

Altogether, the factors mentioned above, the intrinsic heterogeneity of this neoplasia, the invasive capability, the absence of a total tumor resection, and the enormous alterations in the TME, which represent the extrinsic heterogeneity, contribute to tumor resistance (Abou-Antoun et al., 2017; Arbab et al., 2017; Roos et al., 2017).

Regarding TME, multiple factors are known to influence the tumor aggressiveness, mainly hypoxia, the hypervascularity in the microenvironment, and the crosstalk between GBs and GAMs or astrocytes (Markovic et al., 2005; da Fonseca et al., 2016; Hambardzumyan et al., 2016; Audia et al., 2017; Matias et al., 2017b; Guan et al., 2018). In this sense, the next section of the review will explore the role played by microglial and astrocytes cells in modulating GB malignancy and therapy resistance.

## Microglia and Astrocytes’ Role in Glioblastoma Malignancy

This might be one of the most exploited topics regarding TME, and it plays the main role during tumor progression, influencing cancer invasion, aggressiveness, and resistance (Pitt et al., 2016). In particular, GBs are recognized as heterogeneous tumors due to the presence of different subpopulations within the tumor mass, some of them with distinct origin of neoplastic cells. This heterogeneity is established by the interaction between tumor cells and “stroma cells,” such as astrocytes and microglial cells, which are normally responsible for the brain homeostasis (Hambardzumyan et al., 2016; Gieryng et al., 2017b; Roos et al., 2017; Roesch et al., 2018). Nevertheless, in the context of the tumor, the glial and immune cells do not recognize the cancer cells as an intruder, and so do not fight them. So how do the tumor cells evade the immune system?

According to the histopathological and flow cytometry studies, the human and mice glioma biopsies are composed of 30–40% of microglial cells (Graeber et al., 2002; Garcia et al., 2014; Huszthy et al., 2015; Annovazzi et al., 2018). Microglia is called the glial cell; however, their origin is distinct from the other glial cells. The microglial cells are derived from the precursors of yolk sac during early embryogenesis instead of radial glia-like astrocytes (Del Rio Ortega, 1932; Ginhoux et al., 2013).

In fact, the microglial cells are considered the immune cells of the CNS (Del Rio Ortega, 1932; Ginhoux et al., 2013) since they share macrophages characteristic of acquiring different phenotypes depending on the pathological stimuli. For many years, it has been considered that the M1/M2 polarization that occurs in macrophages are also present in microglial cells. These phenotypes have been associated with the distinct production of pro-inflammatory or anti-inflammatory cytokines (Hambardzumyan et al., 2016; Lisi et al., 2017). Although these cells have a notable plasticity, and some studies showed that microglial cells could converge between M1/M2-like phenotype (Hambardzumyan et al., 2016; Lisi et al., 2017; Matias et al., 2018), they can also display different protein expressions of both M1- and M2-like phenotypes at the same time (Lisi et al., 2014; Gabrusiewicz et al., 2016; Ransohoff and El Khoury, 2016). The expression of pro-inflammatory cytokines, such as IL-2 and 12, interferon gamma (IFN-γ), and TNF-α, has been associated with the M1-like phenotype. Meanwhile, the presence of IL-10 and TNF-β is apparently associated to the M2-like phenotype (Roessler et al., 1995; Hambardzumyan et al., 2016; Lisi et al., 2017). However, other proteins have been associated with the M1/M2 polarization of microglial cells. For example, regarding the microglial cells with M1-like phenotype, the increased levels of cytokines and ILs, such as TNF-α and IL-12, IL-23, and IL-1β, as well as NO, were normally observed. In parallel, the M2-like phenotype not only presents high expression of IL-10, but also increased levels of ARG-1, stress inducible protein (STI-1), and inducible NO synthase (iNOS) (da Fonseca et al., 2016; Hambardzumyan et al., 2016; Annovazzi et al., 2018). Also, GAMs can express some markers, such as cluster of differentiation 163 (CD163), a marker highly specific for the M2-like phenotype (Lau et al., 2004), and CD68 that is expressed by both M1 and M2 macrophages (Holness and Simmons, 1993). Recently, it has been described that the transmembrane protein 119 (TMEM119) can also be used as a specific microglia marker (Satoh et al., 2016).

In the case of GBs, glioma cells are able to suppress the microglial M1-like phenotype and induce an M2 anti-inflammatory phenotype through the above-mentioned cytokines and chemokines, which in turn induce microglial cells to release different factors that will stimulate the tumor growth (Hambardzumyan et al., 2016; Lisi et al., 2017). Moreover, the analysis of brain slices from a rat model of glioma using two-color time-lapse fluorescence microscopy showed that glioma cells induce microglia motility (Juliano et al., 2018). The transcriptome analysis of GBs showed upregulation of genes that are involved in the invasion capability and immunosuppression (Mieczkowski et al., 2015; Gieryng et al., 2017a).

In fact, the immunosuppressive microenvironment in GB occurs in various circumstances, which include the action of the GAMs and myeloid-derived suppressor cells (MDSCs) (Chang et al., 2016; Gieryng et al., 2017a). In the case of GBs, glioma cells are able to stimulate GAMs to produce immunosuppressive molecules, such as IL-10, metalloproteinases (MMPs), chemokines as monocyte chemotactic protein-1 (CCL2), and ARG-1, that stimulate the tumor growth (Chang et al., 2016; Zhang I. et al., 2016; Gieryng et al., 2017b; Lisi et al., 2017). Moreover, the presence of microglial M2-like phenotype has been associated with the aggressiveness and poor prognosis in GB patients (Mieczkowski et al., 2015; Iglesia et al., 2016; Gieryng et al., 2017a). Recently, it has been shown that CCL2 released by GB and GAMs acts as a chemoattractant to C–C chemokine receptor type 2 (CCR2) + Ly-6C + MDSC (Chang et al., 2016). Moreover, MDSCs have the ability to release important cytokines, to name a few, the TGF-β and IL10, among others, inside solid tumors, acting as immunosuppressants and promoting tumor growth. In this sense, inhibiting MDSCs function has been shown to promote antitumor immune responses (Zhang et al., 2018).

Moreover, tumor-directed cytotoxic T lymphocytes function is also regulated by immune checkpoints, such as programmed death (PD-1) receptor and their ligands PD-L1 and PD-L2 (DiDomenico et al., 2018). Specifically, PD-L1 is highly expressed in tumors, including gliomas. PD-L1 functions through its interaction with correspondent receptor PD-1 in the activated T cells. From this interaction results an inhibitory response. In this sense, increased expression of PD-L1 in GB patients has been correlated with poor survival of those receiving vaccine immunotherapy (DiDomenico et al., 2018). On the other hand, GAMs also release PD-L1 and, consequently, block the T cells through PD-1/PD-L1 signaling (Preusser et al., 2015).

There are different types of glial cells with different origins and ontogenies, for example, the astrocytes, as previously mentioned. In fact, other studies have shown that tumor cells can manipulate the behavior of astrocytes; by releasing a variety of factors, such as IL-10 and interferon beta (IFN-β), can also stimulate the anti-inflammatory phenotype of these cells (Guan et al., 2018). Recently, a study demonstrated that depending on the subpopulation of astrocytes submitted to the same oncogenic mutation (oncogenic TRP mutations), different types of gliomas could arise. For instance, the transformed glial fibrillary acidic protein (GFAP)-positive astrocytes induced anaplastic astrocytomas (WHO grade III and IV), while the transformed glutamate/L-aspartate transporter (GLAST)-positive astrocytes formed low-grade astrocytomas (WHO grade I) (Irvin et al., 2017).

Thus, as described above, microglia and astrocytes appear to be involved in various mechanisms connected to tumor growth. Here, we will describe recent discoveries about the factors that manipulate or may be involved in the interaction between tumor cells and entities from TME, such as microglia and astrocytes.

### The Multiple Factors Involved in Microglia/Astrocytes–Glioblastoma Interactions

**FIGURE 1:**
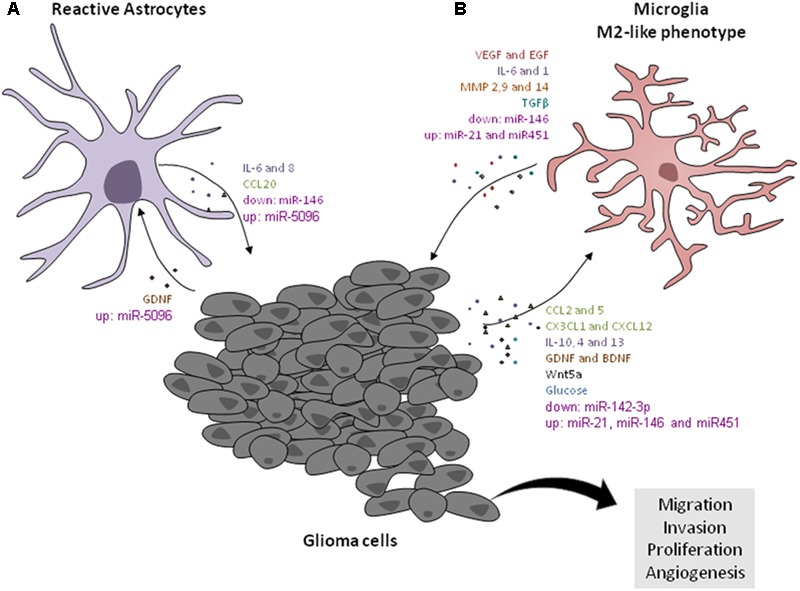
Factors involved in microglial/astrocytic– GB cells crosstalk. **(A)** Astrocytic cells release IL-6 that contributes for tumor migration and invasion through the release of MMP14s by tumors cells. The astrocytic cells released CCL20, which increases HIF-1α in GB cells via CCR6/NF-êB activation. GDNF released, by glioma cells, also promotes astrogliosis. GB cells transfer miR-5096 to astrocytes. **(B)** The GB cells release interleukins (IL10, IL4, and IL13) that induce the microglial cells to acquire M2-like phenotype. In turn, microglial cells secrete TGF-β that stimulate the glioma growth. The invasion and migration of tumor cells are stimulated by the MMPs production by microglial cells. The tumor cells release the CCL2, which stimulates the release of IL-6 by microglia, and consequently induce the invasion of tumor cells. Also CCL5, produced by the GB cells, increases gene expression and function of the M2 markers ARG-1 and IL-10 on microglial cells. In addition, the GB cells express chemokine CX3CL1, which promotes the recruitment of microglial cells, through its receptor CX3CR1, and increases the expression of MMPs 2, 9, and 14, in the tumor cells, promoting tumor invasion. GDNF secreted from gliomas acts as chemoattractant for microglia. The IL-6 produced by microglia stimulates the expression of VEGF in tumor cells. Also, microglial cells release VEGF-A in tumor hypoxia areas. Immune brain cells express EGF in the tumor border, and thus stimulate invasion. Wnt5a released by glioma cells induces the M2-like phenotype in microglial cells through the increase of CX3CR1, CX43, CD163, and IBA-1. Glioma-released glucose induces the expression of GLUT5 in microglia and contributes to tumor growth. The downregulated levels of miR-142-3p are associated with M2-like phenotype in GAMs. IL-1 induces upregulation of miRNAs in gliomas involved in inflammation, such as miR-21 and miR-146. MiR-146 is downregulated in microglial cells and is associated with glioma growth. miRNA-21 and miRNA-451 are overexpressed in GB and are transferred to microglial cells.

There are several factors implicated in the microglia/astrocytes–GB crosstalk. Among them, alterations on extracellular matrix (ECM) components, cytoskeletal rearrangements, the cytokines, the chemokines, the neurotrophic and morphogenic factors, the metabolic factors, and the non-coding RNAs regulation are the most important ones and will be described in detail in the “The multiple factors involved in microglia/astrocytes–glioblastoma interactions” section. The factors involved in microglia/astrocytes–GB interactions are represented in **Figure 1**.

#### Extracellular Matrix and Cytoskeletal Modulation During Microglia Polarization With M1/M2-Like Phenotype and Reactive Astrocytes

During the inflammation process, there occur ECM and cytoskeleton rearrangements in TME. It has been shown that microglial cells are recruited to GBs by acquiring invasive and migration abilities (Hambardzumyan et al., 2016; Wang et al., 2017). Microglia migration and invasion capacity depend on their M1/M2 polarization. In IL-4-treated microglial cells, a higher migratory capacity through lower lamellipodia and extension of membrane ruffles was observed, and it accompanied a reduction of cell adhesion compared to lipopolysaccharides (LPS)-stimulated microglial cells (Lively and Schlichter, 2013).

Indeed, the process of changing microglia cytoskeletal occurs through the reorganization of microtubules involving the tubulin B cofactor (TBCB), which confers different microglia phenotypes, as described above (Fanarraga et al., 2009).

In parallel, recent studies have shown altered effects in the contraction of actin filaments on microglia functions. Microglial cells acquire a phagocytic phenotype through triggering receptor expressed on myeloid cells 2 (TREM2) engagement, and consequently, the F-actin rearrangements occur (Forabosco et al., 2013).

Specifically, the reduction of the actin filament is related to cellular senescence resulting in the decrease of proliferation, migration, and phagocytosis associated to microglia events, as well as the M2 polarization (Uhlemann et al., 2016).

In addition to the cytoskeletal changes, ECM proteins also participate in this regulation. Xia et al. (2016) showed that tenascin-C (TNC) produced by GB cells could modulate the phenotype of microglial cells. In fact, the TNC knockdown, in GB xenografts, showed that major histocompatibility complex (MHC) class II^+^/ionized calcium binding adaptor molecule 1 (IBA1)^+^ immune cells presented different morphology involving fewer extensions. These alterations represent some activated amoeboid-like microglia in the center of the tumor mass compared to control GB xenografts, which presents a ramified microglia, a typical phenotype that is also observed in normal brain tissue (Xia et al., 2016).

Moreover, Ellert-Miklaszewska et al. (2016) demonstrated that osteopontin and lactadherin are released by GB cells and induce the M2-like phenotype on microglial cells by transforming those cells into amoeboid, ultimately increasing their ARG-1 levels. Together, these authors also observed that osteopontin and lactadherin derived from GB cells activate integrin/FAK/PI3K signaling pathway on microglial cells, promoting the rearrangements of the actin cytoskeleton, and consequently inducing the migration of the microglial cells (Ellert-Miklaszewska et al., 2016).

Ultimately, the Fibulin-3 is an extracellular protein described to be released by GB cells, and its expression is associated with tumor invasiveness and proliferation. Besides, an *in vivo* study showed that in the core and border of fibulin-3-overexpressed GB occurs the activation of NF-κB in p65+ astrocytes and GB cells (Nandhu et al., 2017).

Therefore, ECM and cytoskeletal proteins may be related to the crosstalk between microglia/astrocytes and GB. However, how cytoskeletal changes occur (e.g., intermediate filaments) and what types of ECM proteins are involved (such as laminin and collagen) in this crosstalk needs to be better explored.

#### Cytokines

Glioblastoma casts an anti-inflammatory cytokine profile by producing vascular endothelial growth factor (VEGF), IL-10 and IL-6, and TGF-β (Jackson et al., 2011).

Transforming growth factor beta is a cytokine that has a dual and complex role in cancer progression, depending on the stage of tumorigenesis and cell type (Landskron et al., 2014). In early stages of tumorigenesis, TGF-β is a strong inhibitor of astrocytes, epithelial, and immune cell proliferation, by promoting apoptosis and inhibiting cell-cycle progression (Joseph et al., 2013; Landskron et al., 2014). This occurs mainly because of the down-regulation of c-Myc and upregulation of cyclin-dependent kinase inhibitor (CKI) P21 (Malliri et al., 1996). However, in later stages, TGF-β increases metastasis and tumor invasion by inducing glial–mesenchymal transition (GMT) (Morrison et al., 2013). Therefore, TGF-β is a cytokine also involved in glioma initiation, migration, and infiltration (Derynck et al., 2001; Wick et al., 2001; Roberts and Wakefield, 2003).

Microglial cells secrete TGF-β, and its inhibition abrogates glioma growth (Wesolowska et al., 2008). Recently, Zhu et al. (2016) showed that TGF-β levels were twofold higher in microglia treated with glioma-conditioned medium (G/MCM) when compared to that in glioma-conditioned medium (G/CM), which shows that glioma stimulates microglia to produce high levels of TGF-β (Zhu et al., 2016).

In addition, ILs are one of the most important molecules that regulate the immune response in the brain, and one of the most studied is IL-10 (Lobo-Silva et al., 2016). GB suppresses the immune response through IL-10, which is able to downregulate the MHC II expression in microglia, impairing the proliferative response of T cells (Frei et al., 1994).

In this sense, IL-10 acts by downregulating pro-inflammatory cytokines in GAMs, such as IFN and TNF production (Fulton et al., 1998).

Together with the IL-4, IL-13, and glucocorticoid hormones, the IL-10 induces GAMs to acquire M2-like phenotype (Mantovani et al., 2002). GAMs have been related with the glioma progression (Ford et al., 1995; Badie and Schartner, 2000; Coniglio et al., 2012). Cai et al. (2015) identified six cytokine signatures among a group of glioma patients with poor survival prognosis. Additionally, the authors related those cytokines with the M2-like phenotype, such as high levels of mRNA encoding IL-10 and TGF-β1, with the glioma progression (Cai et al., 2015). Altogether, it has been suggested that GAMs release MMPs related to tumor invasion, specifically the MMP-2 and membrane type-1 (MT1)-MMP (Osenkowski et al., 2004; Mayes et al., 2006).

Another cytokine highly regulated in GB samples is IL-6 (Tchirkov et al., 2001). It has been shown that CCL2, produced by tumor cells, increases IL-6 levels released by microglia, which are associated to GB invasiveness and growth in a TLR4-dependent manner (Goswami et al., 1998; Wang et al., 2009; Zhang et al., 2012; a Dzaye et al., 2016). During the tumor invasiveness, IL-6 levels released by microglia increase MMP-9 levels (Yeung et al., 2013).

Interleukin-6 is also responsible for inducing VEGF synthesis, and both cytokines are able to induce tumor angiogenesis (Cohen et al., 1996; Huang et al., 2004).

The pro-angiogenic cytokine, VEGF, has been correlated with a poor prognosis and GB recurrence (Turkowski et al., 2018). GBs are recognized as highly angiogenic tumors (Brem et al., 1972). Besides GBs, GAMs are also able to produce VEGF (Chen et al., 2014; Brandenburg et al., 2016). In fact, Osterberg et al. (2016) demonstrated that VEGF is expressed not only in glioma blood vessels but also in tumor areas enriched with CD14+/CD68+ immune cells. Moreover, they also observed that in glioma hypoxia areas, the GAMs are responsible for VEGF-A production (Osterberg et al., 2016). Interestingly, it has been described that the inhibition of VEGF together with angiopoietin-2 (Ang-2) induce the polarization of GAMs to M1-like phenotype, as well as increase mice survival and delay the tumor growth (Kloepper et al., 2016). On the other hand, a decrease in GAMs infiltration was observed, followed by augmentation of apoptosis and MHC expression in VEGF-overexpressing gliomas, suggesting a regulatory feedback mechanism (Turkowski et al., 2018).

Overall, microglial cells are not the only glial cells that release IL-6, astrocytes too expressed high amounts of IL-6, in particular when co-cultured with GB cell lines, contributing to tumor migration and invasion through the release of the active form of MMP-14 by GB cells (Chen W. et al., 2016).

In this manner, cytokines represent crucial factors involved in microglia/astrocytes–GB crosstalk as represented in **Figure 1**.

#### Chemokines

The chemokines are highly expressed by microglia and astrocytes and are mainly important to their recruitment and activation in cancer (Hattermann and Mentlein, 2014; Laudati et al., 2017; Jin et al., 2018).

Chemokine axes, such as fractalkine (CX3CL1)/CX3CR1, C-X-C motif chemokine ligand 12 (CXCL12)/C-X-C chemokine receptor type 4 and 7(CXCR4/CXCR7), CCL2/CCR2, and CCL5/CCR5, are clearly involved in tumor progression (Held-Feindt et al., 2010; Hattermann and Mentlein, 2014; Laudati et al., 2017).

Glioma-associated microglia/macrophages are characterized by the expression of those chemokines or receptors (Hattermann et al., 2014). And if the expression of GAMs M2-markers is decreased, this significantly increases survival and suppresses the growth of established tumor (Pyonteck et al., 2013).

In the brain, the neurons and astrocytes produce CX3CL1, and the receptor is restricted to microglia (Ludwig and Mentlein, 2008; Ransohoff and El Khoury, 2016). The CX3CL1 expressed by GB cells induces the human GAMs recruitment through its receptor CX3CR1 and increases the expression of matrix MMPs 2, 9, and 14 in GAMs, which consequently promotes the tumor invasion (Held-Feindt et al., 2010; Carvalho da Fonseca and Badie, 2013; Ferrer et al., 2018). In humans, but not in rodent models, the prolonged average of survival time of glioma patients and the low infiltration of microglia is associated with the CX3CR1 gene (Liu et al., 2008; Rodero et al., 2008).

In turn, CXCL12 is widely produced in gliomas and normal brain. In tumor areas with necrosis, angiogenesis, and invasion, the GSCs express mainly the CXCR4 (Hattermann and Mentlein, 2014). ALTS1C1 cells, a new murine glioma model, expressed a relatively high level of CXCL12 *in vitro* and *in vivo*. The inhibition of CXCL12 by a specific siRNA has reduced tumor infiltration, and the formed tumors had defined borders. In addition, the inhibition of CXCL12 synthesis reduced migration of GAMs toward hypoxia regions (Wang et al., 2012). Therefore, the alteration of CXCL12-concentration gradient could be an important approach for the tropism of GAMs toward hypoxia areas (Hattermann and Mentlein, 2014). In the last decade, several preclinical studies in glioma models have shown that the impairment of the CXCL12/CXCR4 signaling using specific antagonists reduces tumor growth, angiogenesis, and recurrence, suggesting that this approach represents a promising strategy for GB therapy (Tseng et al., 2011; Liu et al., 2014).

The CCL2 was firstly detected in glioma cell supernatants and confirmed to be a chemoattractant protein for immune cells *in vitro* (Yoshimura et al., 1989). The astrocytes and microglia are the main sources of CCL2 in CNS (Vakilian et al., 2017). In GBs, the CCL2 expression is correlated with GAMs by recruitment of regulatory T cells and MDSCs to increase GB growth (Hattermann and Mentlein, 2014; Chang et al., 2016). Specifically, the glioma growth is induced by the IL-6 levels, which are produced by microglial cells under stimulation of glioma-released CCL2 that acts upon CCR2 in microglia. The CCL2–CCR2–IL-6 pathway is considered a potential therapeutic target for gliomas (Zhang et al., 2012). In addition, an interesting study demonstrated that astrocytes enhance the invasion of CD133+ GSCs, providing a microenvironment rich in cytokines and chemokines, such as CCL2 (Rath et al., 2013).

In addition to the chemokines mentioned above, the amounts of CCL5 and CCR5 expression are correlated with poor prognosis and average survival time of GB patients (Laudati et al., 2017). Moreover, GAMs produce CCL5 and, consequently, potentiate the regulation of neurofibromatosis type 1 (NF1), associated with a murine low-grade optic glioma growth (Solga et al., 2015). CCL5 produced by the GB cells acts by increasing its cell survival using CD44 as a receptor, to suppress programmed cell death (Pan et al., 2017). Interestingly, Laudati et al. (2017) found that maraviroc (MRV), a CCR5 blocker, reduced the M2-like markers ARG-1 and IL-10 levels and function. In addition, the inhibition of CCR5 was correlated with a significant decrease of microglia migration, provoked by the inhibition of PI3-AKT pathway (Laudati et al., 2017).

It is already known that astrocytes–GB interactions can occur through the CCLs and ILs. In hypoxic conditions, the astrocyte-released CCL20 increases hypoxia-inducible factor 1-alpha (HIF-1α) in GB cells via CCR6/nuclear factor kappa B (NF-κB) activation and, in turn, induces GB cells to adapt to hypoxia (Jin et al., 2018).

Taken together, the communication network established among gliomas cells with TAMs and astrocytes cooperates through the chemokines (**Figure 1**) to increase glioma growth and infiltration into brain parenchyma, contributing to poor patient survival, and therefore, blockage of these pathways could assist in better therapy options for these patients.

#### Neurotrophic and Morphogenic Factors

During the last few years, the neurotrophic factors (NTFs) have gained importance due to their function of not only growth, differentiation, and survival of both mature and developing neurons (Ku et al., 2013; Liu et al., 2013) but also in modulation of microglial cells and astrocytes activity (Tanaka et al., 2008; Xu et al., 2018). The NTFs could be divided into six families (**Table 1**).

**Table 1:** Neurotrophic and morphogenic factors involved in modulation of microglia/astrocytes phenotype in GB context.

Family		Type of factors	Hallmarks	References
Neurotrophic factors	Neurotrophins	Brain-derived neurotrophic factor (BDNF)	The BDNF derived from the brain-stimulated microglial cells to produce IL-15 and consequently increase NK cell infiltration and activation, contributing to limit glioma expansion.	(Garofalo et al., 2017)
		Nerve growth factor (NGF)	NGF is expressed by glioma cells. Microglia expresses NGF receptors and when exposed to NGF, acquire neuroprotective and anti-inflammatory phenotype through modulation of motility, phagocytosis, and degradation pathways. No data in microglia–GB crosstalk.	(Lawn et al., 2015; Rizzi et al., 2018)
		Neurotrophin-3 (NT-3)	NT-3 is important for the proliferation of brain tumor-initiating cells. Microglial cells express NT-3. No data in microglia–GB crosstalk.	(Zhang et al., 2009; Forsyth et al., 2014)
	CNTF	Ciliary neurotrophic factor (CNTF)	CNTF is synthesized and secreted by gliomas and inhibits T-cell activation and microglia activation. Microglia express CNTF receptor alpha (CNTFRα) and its stimulation with CNTF induces COX2 expression. CNT and CNTFα are expressed by astrocytes. No data in astrocytes–GB crosstalk.	(Dallner et al., 2002; Lin et al., 2009; Olah et al., 2012)
	GDNF	Glial cell line-derived neurotrophic factor (GDNF)	Microglia and astrocytes express GFRa-1 and GFRa-2. Glioma-released GDNF acts as chemoattractant for microglia and did not induce astrogliosis.	(Ku et al., 2013)
	Ephrins	Ephrin-B3	Ephrin-B3 is highly expressed in GBs. Astrocytes express EphB3/EphA4 receptors. No data in astrocytes–GB crosstalk.	(Zhuang et al., 2011; Royet et al., 2017)
	EGF and TGF	Transforming growth factor beta (TGFβ)	Reactive astrocytes have shown to secrete TGF-β, which increases the proliferation and invasion of tumor cell.	(Zhang et al., 2015)
		Vascular endothelial growth factor (VEGF)	High levels of VEGF induce GB progression and a marked remodeling of the vascular structure accompanied with an important reduction of microglial cells.	(Turkowski et al., 2018)
	Others	Macrophage colony-stimulating factor (M-CSF)	M-CSF [also named colony-stimulating factor 1 (CSF-1)] promotes the upregulation of several M2 markers and thereby actively contribute to the M2-like GAM polarization.	(Komohara et al., 2008)
		Granulocyte–macrophage colony-stimulating factor (GM-CSF)	Microglia and astrocytes express GM-CSF receptor, GM-CSF induces an inflammatory response through IL-1β, TNFα, IL-10, and IL-6 increased levels. GM-CSF is secreted by glioma cells and induces GAM (IBA1+ cells) invasion.	(Sielska et al., 2013; Nicol et al., 2018)
Morphogenic factors	Hedgehog	Sonic Hedgehog (Shh)	Reactive astrocytes-released Shh drives proliferation of IBA1+ immune cells (microglia/macrophages). LPS-treated microglial cells induce Shh expression. GB also secrete Shh. No data in microglia/astrocytes–GB crosstalk.	(Pitter et al., 2014; Rimkus et al., 2016; Lee et al., 2017)
	Wingless-type MMTV integration site	Wnt5a	GB-released Wnt5a increase infiltration of the immune cells (microglia). No data in astrocytes–GB crosstalk.	(Dijksterhuis et al., 2015)
	family (Wnt)	Wnt3a	Microglial cells and astrocytes express Frizzled (FZD) and LRP5/6 receptors. Wnt3a is highly expressed in GB cells. No data in microglia/astrocytes–GB crosstalk.	(Halleskog et al., 2011; Denysenko et al., 2016)

Moreover, it has been reported that glial-derived neurotrophic factor (GDNF) receptors, namely, GFRa-1 and GFRa-2, are expressed by microglial cells (Honda et al., 1999; Rickert et al., 2014). Ku et al. (2013) showed that the GDNF secreted from gliomas acts as a chemoattractant for microglia. In this study, the authors showed that mice xenografts of glioma cells silenced to GDNF reduced the GAMs and generated smaller tumors. Additionally, the GDNF released by glioma cells also promotes astrogliosis; in spite of the downregulation of GDNF, they did not observe any differences in astrocytes that surrounded the tumor mass (Ku et al., 2013). Besides that, GB, as well as microglial cells, secretes cytokine TGF-β, which is important to stimulate tumor cell invasion (Han et al., 2015; Kim et al., 2017). Furthermore, TGF-β in the presence of oncolytic herpes simplex viruses (oHSV) plays an anti-inflammatory role during glioma progression by decreasing the percentage of immune cells (CD45^+^CD11b^+^) and, consequently, reducing their NO synthase 2 (NOS2) and TNF-α mRNA and protein levels involved in M1-like phenotype on microglial cells (Han et al., 2015). On the other hand, GSCs become more invasive, thereby promoting glioma progression in response to the TGF-β1 secreted by the CD11b^+^F4/80^+^ immune cell population (Ye et al., 2012).

Moreover, TGF-β can be associated with the VEGF expression, which is important not only for tumor vascularization but also in the modulation of macrophages/microglial cells activity (Roy et al., 2015). Recently, it has been demonstrated that high levels of VEGF induce GB progression and a marked remodeling of the vascular structure accompanied by an important reduction of IBA1^+^ cells. However, less accumulation of IBA1^+^ cells at the perivascular niche induced a reduction of pro-angiogenic factors (such as VEGF) released, suggesting a possible regulatory feedback mechanism (Turkowski et al., 2018). The authors also suggested that during an anti-VEGF therapy and standard VEGF expression, the tumor might manipulate microglia/macrophages to collaborate in angiogenesis (Turkowski et al., 2018).

Other NTF, such as colony stimulating factor-1 (CSF-1) and EGF, are also important to microglia–GB crosstalk (Pyonteck et al., 2013; Komohara et al., 2016; Kim et al., 2017). CSF1 acts as an oncogene by inducing gliomagenesis. Moreover, it was observed that the periphery of tumor mass of high-grade gliomas presents a minority population of GAMs that express ARG1 and CD206, typical markers for M2 polarization (De et al., 2016). Further, the IBA1^+^ and CD163^+^ immune brain cells express EGF in the tumor border and thus stimulate the tumor cells to invade the parenchyma (Komohara et al., 2016; Hide et al., 2018).

Moreover, brain-derived neurotrophic factor (BDNF) is a very interesting NTF in the tumor context. GAMs, stimulated by brain-released BDNF, induced IL-15 production and, consequently, increased NK cells’ infiltration and activation, contributing to limit glioma expansion (Garofalo et al., 2017).

Nevertheless, the morphogenic factors also play important roles in microglia/astrocytes–glioma crosstalk, mainly, the wingless-type MMTV integration site family (WNT) and sonic hedgehog (SHH). During early embryogenesis, these two factors are crucial for proper development of the tissues and organs (Matias et al., 2017b; Carballo et al., 2018). However, these pathways are recapitulated and play crucial roles during tumor development. Moreover, its influence on the immune system has also been highlighted over the last few years (Matias et al., 2017b).

In the TME, it was demonstrated that SHH is important for controlling epithelial–mesenchymal transition (EMT) – through a paracrine and autocrine manner – the pathogenesis, and progression of tumors, such as brain and prostate cancers (Tzelepi et al., 2011; Islam et al., 2016; Ming et al., 2017). In case of brain tumors, a study showed that in SHH subgroup of medulloblastoma, the tumor-macrophages/microglial cells might contribute to tumor growth, which has already been suggested as a potential therapeutic approach (Margol et al., 2015). Besides, it is already known that GB could secrete SHH (Rimkus et al., 2016). Additionally, reactive astrocytes can secrete Shh, inducing the proliferation of microglia, oligodendrocyte transcription factor (OLIG2) positive progenitors, and astrocytes after a neurodegeneration process (Pitter et al., 2014). However, the role of SHH from the GB cells in microglia and astrocytes proliferation and activation remains unclear.

Besides, it has been observed that SHH also interacts with other signaling pathways normally activated in tumors, including brain tumors, such as TGF-β and the canonical WNT pathway (Carballo et al., 2018). It has been suggested that the WNT components can also be regulators of microglia–GB interactions (Matias et al., 2018). Both microglial and astroglial cells express high levels of WNT and FRIZZLED (FZD) receptors and play major roles in the modulation of the inflammatory response and cellular mechanisms, such as proliferation, migration, and invasion (L’Episcopo et al., 2018).

It has been described that during the inflammatory response in different contexts, the canonical and non-canonical WNT signaling pathways – more specifically, WNT3a and WNT5a – have a great relevance (Halleskog and Schulte, 2013; Silva-García et al., 2014; Suryawanshi et al., 2015; Chen K. et al., 2016). Recently, it was suggested that WNT5a plays a crucial role in the interaction of the tumor with microglia. *In silico* studies, using GB samples from the TCGA and immunohistochemistry of human biopsies (TMAs), have shown that gliomas have high expression of WNT5a and that this aberrant expression is associated with the presence of immune cells. Moreover, this study showed that different groups, according to WNT5a expression, have different gene patterns. The glioma groups with high expression of WNT5a presented high levels of CX3CR1, connexin 43 (CX43), CD163, and IBA-1, suggesting that the GB-infiltrating microglial cells are activated, which represent the M2-like phenotype. In addition, signaling pathways associated with the inflammatory response, such as toll-like receptors (TLRs), were identified (Dijksterhuis et al., 2015). Furthermore, it has been suggested that microglia may be one of the signaling entities for breast cancer cell metastases in the brain, since the microglia of organotypic brain cultures produce high levels of Wnt5a, promoting the growth of breast cancer (Pukrop et al., 2010; Nayak et al., 2012).

The glioma microenvironment can promote the activation of reactive astrocytes characterized by high expression of GFAP and the glycoprotein podoplanin. Lu et al. (2016) showed that tumor-associated astrocytes (TAAs) exhibited high-migration and invasion capability through the induction of GMT by increasing vimentin and MMP levels, which are WNT target proteins. Moreover, they observed that WNT/β-catenin signaling pathway is activated in TAAS, suggesting that glioma cells induce the activation of TAAS via WNT/β-catenin signaling (Lu et al., 2016).

Altogether, the NTFs and morphogenic factors may also influence the immune system in tumors and, therefore, could be potential targets for tumor therapies (**Figure 1**). This subject still needs to be further exploited to address the role of these molecules in this crosstalk.

#### Metabolic Factors

In the last decades, some studies tried to understand how the metabolism of the CNS cells behaves in different physiological context, such as inflammation and cancer.

All types of cells need vast amounts of adenosine triphosphate (ATP) to survive, including microglia and astrocytes (Davalos et al., 2005; Engl and Attwell, 2015; Jackson and Robinson, 2018). Depending on the oxygen conditions, cells can produce ATP by the conversion of glucose to pyruvate through the mitochondrial tricarboxylic acid cycle (TCA) and glycolysis. On the other hand, in the absence of oxygen (hypoxia), cells transform the pyruvate into lactate by anaerobic glycolysis (Orihuela et al., 2016; Ghosh et al., 2018). Therefore, the study of the metabolic alterations that occur in microglia and astrocytes can be important in understanding the microglia polarization of M1–M2 and the astrocytes reactivity that occurs in GB.

During the polarization of M1/M2-like phenotypes in microglial cells, some alterations occur in metabolic circuits and mitochondrial respiration (Orihuela et al., 2016). Microglia express different transporters for main metabolites, such as glucose, glutamine, and fatty acids (Ghosh et al., 2018). One of the proteins that regulate the independent transport of glucose in microglia, and also in tumor cells, is the family of glucose transporter (GLUT) (Sasaki et al., 2004). Sasaki et al. (2004) showed that the GAMs express GLUT5 in non-necrotic areas of the tumor. Moreover, the microglial cells, under glucose deprivation (GD), increase the secretion of IL-6 (Choi et al., 2015) as well as their phagocytic behavior (Churchward et al., 2018). Taking into account that IL-6 and phagocytosis have been associated to M1-like phenotype in microglia (Hambardzumyan et al., 2016), the downregulation of glucose levels in GB or even the inhibition of GLUT5 may be useful to induce M1 polarization in microglial cells and, consequently, reduce the tumor aggressiveness.

The energy potential of mitochondria and other organelles for the cellular energy production machinery is indisputable since it participates in different fronts by performing various actions. Among them are the control of cytosolic calcium (Ca^2+^) levels, the control of apoptosis through the transition of pore permeabilization, the reduction–oxidation modulation (redox), and the production of reactive oxygen species (ROS) (Wallace, 2012).

The variation of the internal Ca^2+^ levels, the cellular stress, or the decrease in the number of copies of the mitochondrial DNA can cause alterations of the membrane potential. Normally, the absorption of Ca^2+^ by the membrane of the mitochondria inhibits apoptosis of the cells and also induces high levels of ROS, which consequently leads to neoplastic transformation of the cells. However, high levels of ROS induce cellular apoptosis and necrosis because of its cellular toxicity. The increase of Ca^2+^ levels occurs in the cytoplasm compartment, which induces activation of Ca^2+^ signaling pathways that regulate tumorigenesis and invasive tumor growth (Gupta et al., 2012; Wallace, 2012).

Recently, it has been under discussion that the metabolic alterations contribute to the aggressiveness of the tumors, through the dysregulation of mitochondrial and cytoplasmic enzymes, such as enzyme isocitrate dehydrogenase (IDH) (Al-Khallaf, 2017). Particularly in gliomas, the presence of mutations in this enzyme, which has been associated to better prognosis and survival, comparably to IDH wild-type, has been reported (Louis et al., 2016). Normally, IDH1 is present in the cytoplasm and interfere with the catalyzation of isocitrate (ICT) into α-ketoglutarate (α-KG) to produce nicotinamide adenine dinucleotide phosphate (NADPH) from NADP^+^. Instead, IDH2 regulates the reverse reaction of α-KG into ICT in mitochondria (Al-Khallaf, 2017).

However, the presence of the mutations, *IDH1-R132* and *IDH2-R172*, causes the reduction of α-KG and an increase of R-2-hydroxyglutarate (2HG) levels, an oncometabolite that induces alterations on chromatin and histone methylation (Ward et al., 2010; Koivunen et al., 2012; Amankulor et al., 2017).

How the *IDH* mutations interfere/modulate the TME has also been addressed in the literature. For instance, a study using a cohort of 60 patients with gliomas with *IDH1–R132H* mutation was performed to understand if the GAMs samples also have the *IDH1–R132H* mutation. The authors showed that a population of CD68^+^, IBA1^+^, and CX3CR1^+^ GAMs samples also had the *IDH1–R132H* mutation (Zheng et al., 2012). Moreover, mice with *IDH1–R132H* mutation showed elevated 2-HG and deoxyribonucleic acid (DNA) methylation, as well as reduced immunological characteristics, such as reduced number of microglial cells (Zhang X. et al., 2016; Amankulor et al., 2017).

However, the *IDH* mutations might occur in a subgroup of GB. Turcan et al. (2012) showed that IDH1 mutation modulates the differentiation state by increasing the nestin levels in normal human astrocytes (NHAs). Moreover, these *IDH1* mutant cells produce higher levels of 2-HG, which is normally observed in low-grade brain tumors. According to the literature, the *IDH* mutation in cancer patients is associated with better prognostic and survival rate, suggesting that IDH could be a promising target to reduce the aggressiveness of GB by modulating the astrocytes activity (Turcan et al., 2012).

Thus, we hypothesize that alterations in TCA cycle and glycolysis can induce the impairment of aggressiveness of the tumor and pro-inflammatory response (**Figure 1**) and be visualized as a possible target to establish new therapeutic strategies to combat the gliomas.

#### Microglia/Astrocytes and Glioblastoma: The Role Played by Non-coding RNAs

It is well known that RNA biology plays an important role in the CNS where robust expression of non-coding RNAs (ncRNAs) has been found. The ncRNAs are widely defined as all types of RNA that cannot be translated into proteins due to the lack of open-reading frames (ORFs). They can be small ncRNAs (snRNAs) and long ncRNAs (lncRNAs) (Qureshi and Mehler, 2012; Aprea and Calegari, 2015; Ji et al., 2015; Quan et al., 2017).

The microRNAs (miRNAs) are snRNAs capable of regulating gene expression in cancer cells and associated normal cells (Pitt et al., 2016). Their unique features include regulation of multiple targets, ability to be secreted into the extracellular space, and the communication linking capacity between tumor cells and the TME. Therefore, studying miRNAs concerning the complex interactions between the tumor cells and their microenvironment is necessary for understanding the tumor progression and, thus, the development of new therapeutic strategies (Kros et al., 2014; Kohlhapp et al., 2015; Li and Liu, 2016).

Interestingly, the dysregulation of miRNAs in GAMs has been shown to promote tumorigenesis. For instance, the miR-155 is a well-known regulator of the immune response and is involved with the M1/M2 polarization of macrophages/microglia (Cardoso et al., 2012). In the same context, studies that induce the pro-inflammatory phenotype in cultured microglia using LPS demonstrated an increased expression of miR-155 and miR-146 and a decreased expression of miR-689 and miR-124, of which miR-124 was associated with microglia quiescence (Ponomarev et al., 2013; Caldeira et al., 2014; Cunha et al., 2016). Notably, miR-155 was discovered to be constantly downregulated in immune cells, thus promoting tumorigenesis (He et al., 2009).

Another miRNAs have been found to be downregulated within GAMs, including the miR-142-3p and miR-146b-5p (Xu et al., 2014; Zhang et al., 2017). Consequently, the downregulation of miR-146b-5p was found to promote tumor proliferation and invasion ability (Cai et al., 2015) while the downregulation of miR-142-3p is associated with M2-like phenotype in GAMs. *In vivo* study has shown that the administration of miR-142-3p induced the polarization of GAMs into M1-like phenotype, and consequently, the GB growth was reduced and the median survival rate increased (Xu et al., 2014).

Elsewhere, IL-1-induced miRNAs involved in inflammation, such as miR-21 and miR-146, have been found to be upregulated in low-grade gliomas (Tili et al., 2011; Prabowo et al., 2015).

In line with the previous thoughts, characterization of miRNA at the TME border can help to improve prognosis in GB patients. A miRNA expression study conducted using samples from the surrounding tissue of GB mass showed that miR-338-3p, miR-219-2-3p, miR-27b, miR-23b, and miR-219-5p were upregulated while miR-H18, miR-1246, miR-1181, miR-3195, miR-630, miR-642b, and miR-3663-3p were downregulated in the tumor border. Subsequently, miR-338-3p, miR-219-5p, and miR-219-2-3p were correlated to oligodendrocyte differentiation. The number of oligodendrocyte lineage cells was high in the border where macrophages and microglia were also colocalized. Moreover, it was observed in tumor mass border that increased levels of EGF, heparin-binding EGF-like growth factor (HB-EGF), IL-1β, and acidic fibroblast growth factor (FGF1) that correspond to the presence of OPCs and macrophages/microglia are responsible for inducing stem-like phenotype and resistance in GB cells (Hide et al., 2018; **Figure 1**).

**FIGURE 2:**
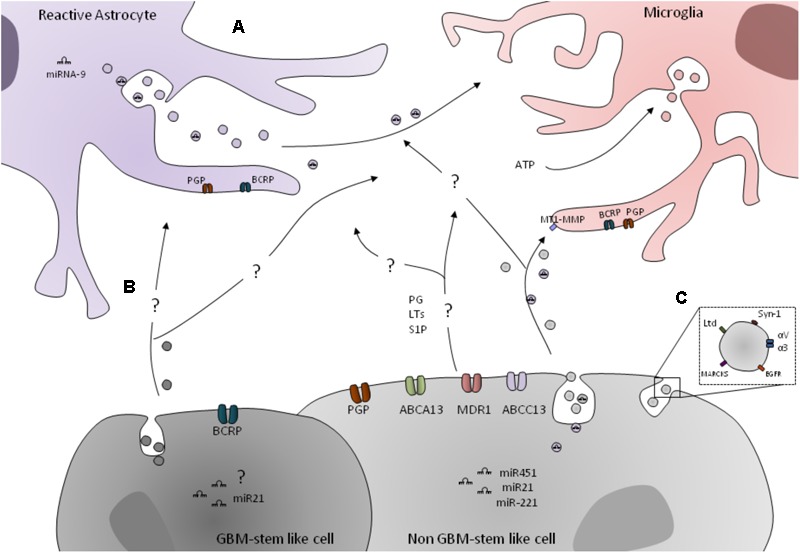
Transportation mechanism involved in glial cells and GB crosstalk. **(A)** GB cells express several ABC transporters, such as ABCB1, ABCC1, ABCG2 and ABCC13, and ABCA13. GB cells express prostaglandins (PG), leukotrienes (LTs), and sphingosine-1-phosphate (S1P), which can be ABS transporters subtracts in microglial/astrocytic cells. The astrocytes and microglial cells express ABCG1 and ABCG2. Other mechanism of cell–cell communication is through the EVs, such as exosomes. GB miRNAs, namely the miR451, miR21, and miR-221, can be transferred through EVs to microglial cells. However, how can they be transferred to astrocytes is still unclear. Also, EVs are a mechanism for GB to induce MT1–MMP expression in GB-associated microglia. Moreover, extracellular ATP promotes microglia activation and induces the release of EVs in order to enable the communication between microenvironment cells. In fact, miR-9 released from Tat-stimulated astrocytes can be taken up by microglial cells, which result in their migratory phenotype. **(B)** ABCG2 is only expressed in GB-stem-like cells. Also, GB-released EVs can transport miRNAs, such as miR2. However, their role in astrocytic and microglial cells is not exploited. **(C)** Lactadherin (Lyd), syntenin-1(Syn-1), myristoylated alanine-rich C-kinase substrate (MARCKS), integrin alpha-V and alpha-3 (αV and α3), and EGFR can be detected in the membrane of GB-released EVs.

Moreover, in GBs, many well-known miRNAs that are highly expressed (let-7a, miR-16, miR-21, miR-27a, miR-26a, miR-93, miR19b, miR-320, miR-20, miR-15b, and miR-92) in the microvesicles are isolated from patients. In this sense, an analysis of miRNAs transference through GB-isolated EVs to microglial cells showed that microglia had taken up these EVs, which increased the levels of miR451/miR21 to 15-fold (van der Vos et al., 2016). This means that probably miRNAs that are overexpressed in GB can be transferred to microglial cells by EVs (**Figure 2**).

Parallel to this, microRNAs can also be transferred between different cells in the TME through the gap junctions. Astrocytes have been shown to form gap junctions with glioma cells (Zhang et al., 1999), which then can potentially assist in tumor invasion. This was demonstrated by a study that found out that the glioma invasion was reduced by the gap junctions formed between glioma cells, while glioma-astrocyte and astrocyte-astrocyte gap junctions promote glioma invasion, at least in part, by the glioma-transferred miR-5096 to astrocytes (Hong et al., 2015).

In addition to its function in microglial cells as described above, the miR-146 is also a negative regulator of astrocyte-mediated inflammation (Iyer et al., 2012). Upregulation of this miRNA has been correlated with a reduced expression of its target, the TNF receptor associated factor (TRAF6), which is involved in a number of seizures in glioma patients. This suggests that miR-146 could be involved in the epileptogenic focus surrounding the tumor (Prabowo et al., 2015).

These studies support the role played by non-coding RNAs in facilitating intercellular communication between immune cells and GB, although their precise contribution in modulating microglia/astrocytes activity in the context of GB still has to be fully elucidated.

### Mechanisms of Transportation and Communication Between Microglial/Astrocytes–Glioblastoma Cells

In order to make possible the microglia/astrocytes–GB crosstalk, some important mechanisms of transportation and/or communication among those cells play an utmost role, to name a few, the ATP Binding Cassette transporters (ABC transporters) and the EVs. We will describe, below, in further detail, the most important mechanisms of transportation and communication among microglial/astrocytes–GB cells, and it is also summarized in **Figure 2**.

#### ABC Transporters

The ABC transporter proteins are a superfamily of proteins, located in biological membranes of most cells from varied species, whose role is the active transport of a wide range of substances, most notably the efflux of endo- and xenobiotics (Johnson and Chen, 2017). These transporters are overexpressed in many cancers, including gliomas, and are responsible for the effluxion of a varied repertoire of chemotherapeutic drugs from cancer cells. The 49 currently known human ABC transporters have been grouped into seven families, ranging from ABCA to ABCG, depending on their sequence homology (Begicevic and Falasca, 2017).

In glioma, the most relevant ABC transporter proteins are p-glycoprotein (ABCB1), MDR-associated protein-1 (ABCC1), and breast cancer resistance protein (ABCG2) (Quezada et al., 2011). Studies have shown that the ABCC1, ABCB1, and ABCA13 are correlated with the level of glioma aggressiveness, with these proteins being overexpressed in high-grade glioma compared to low-grade astrocytoma (de Faria et al., 2008; Balça-Silva et al., 2017a; Dréan et al., 2018).

Despite their role in cancer, the expression of the transporters mentioned above has been documented in other TME cells, mainly in capillary epithelial cells, which constitute BBB. However, there is much less information on brain parenchymal cells, such as microglia, oligodendrocytes, and astrocytes (Gibson et al., 2012). One study that sought to link immune modulators and expression of ABC transporters was conducted in a BV-2 mouse model for microglia function (Gibson et al., 2012). Resting microglia phenotype, in a non-diseased brain, was shown to express mRNAs for transporters ABCC1, ABCC4, ABCB1, and ABCG2 with comparatively low ABCC5. When *in vitro* activation of microglia was performed using LPS, the microglial cells had a profound change in transporter expression with ABCC4, ABCB1, and ABCG2 decreasing half-fold while ABCC1 and ABCC5 had a 140 and 210% increase, respectively (Gibson et al., 2012).

Another study to determine the effect of specific cytokines on transporters was carried out using IL-6 and TNF-α, two proinflammatory cytokines, which are also produced by activated microglia. In primary microglia cell cultures, administration of these cytokines elicited opposing effects on ABCB1 expression, inducing a slight decrease in its mRNA levels after IL-6 administration and a significant increase after TNF treatment at both the gene and protein levels (Shi et al., 2012).

Additional roles are emerging for ABC proteins during the interactions of cancer cells with TME entities. The transportation of molecules into the TME, such as signaling lipids with established roles in tumor biology (for instance, prostaglandins, leukotrienes, and sphingosine-1-phosphate or SIP), and that are known to have profound effects on immune cells (for instance, microglia/macrophages), have been shown to be ABC transporter substrates (Cole, 2014; Fletcher et al., 2016). This raises the question of whether ABC transporter can mediate the efflux of these molecules and how is it important in the context of cancer and, in particular, cell–cell communication of populations within the tumor mass. There are very few studies that have sought to unravel the role played by ABC transporters in the interactions between gliomas and microglia or astrocytes.

Comparative analysis of ABCG2 expression in primary astrocytes and GSCs showed that ABCG2 is only expressed in GSCs and not in normal astrocytes (Chen et al., 2017).

Recently, Dréan et al. (2018) performed a study to verify the expression of ABCA1, ABCA4, ABCA13, ABCC1, ABCC12, ABCC13, and ABCG1 in cohort of 51 newly diagnosed GB patients. They observed that GB overexpression of ABCA13, ABCC1, and ABCC13 are correlated with GB patient progression-free survival. Moreover, they also analyzed the ABC transporters in GB bulk cells, namely, astrocytes and microglial cells. They showed that ABCG1 and ABCG2 are mainly expressed by astrocytes while microglial cells have high expression of ABCG2 compared to the levels of ABCG1, but no ABCA13 expression was observed in these cells (Dréan et al., 2018). However, the differential relation and role of the ABC transporters in these cells is not completely elucidated (**Figure 2**).

So far, the experiments carried out have only shown effects of immune modulators secreted by activated immune cells like microglia/macrophages on the expression of ABC transporters. The overall effect of transporter upregulation or downregulation on the interactions between microglia or astrocytes and glioma, especially those effects that lead to tumor progression, are still under investigation.

#### Extracellular Vesicles

The cells can produce EVs that are constituted by a membrane-limited vesicle that can be divided into three subclasses: apoptotic bodies, microvesicles (MVs), and exosomes (Ciregia et al., 2017).

The differences between all the three EV types are dependent and based on the origin and size of each one. The largest EVs, normally observed in cell death mechanisms, have a size that varies between this 1,000 and 5,000 nm. The MVs derived from the external budding of the membrane can vary between 100 and 1,000 nm; and finally, the smallest vesicles (30–100 nm) are the exosomes (Ciregia et al., 2017).

Among the EVs, the exosomes are the best described and enabled via communication between cells involved in many physiological processes, including its involvement in pathological condition by activating signaling pathways and changing components of the membrane and its neighbor cells (Abels and Breakefield, 2016; Ciregia et al., 2017).

Almost all the body fluids of humans [i.e., cerebrospinal fluid (CSF), blood plasma, saliva, or urine] have exosomes, so they are considered a promising diagnostic and prognostic biomarkers (Akers et al., 2015).

Regarding brain tumors, GB-derived MVs represent one of the mechanisms by which cancer cells can modulate the TME to a more permissive condition for growth and invasion (Dubois et al., 2014).

Our group has recently shown the plasticity of the stem-like cell state through the interconversion of more differentiated cells into a dedifferentiated state (Balça-Silva et al., 2017b). In fact, other authors mentioned that this plasticity could be mediated by MVs in *in vivo* conditions (Quesenberry et al., 2015).

In parallel, the downregulation of mRNA expression levels of TIMP metallopeptidase inhibitor 1(TIMP1), TGF-β, and IL-8 in exosomes is correlated with the survival of GB patients after a vaccination with dendritic cell-derived exosomes, which highlights a potential role of EVs in glioma immunotherapy (Muller et al., 2015).

Regarding the miRNAs’ importance in gene regulation, it is also known in literature that several microRNAs are found to be encapsulated in EVs in the serum of glioma patients, which make them potential circulating biomarkers for early diagnosis, tumor staging, and prognosis. In fact, in serum-derived exosomes of GB patients and CSF-derived EVs, the miR-21 and miR-221 were shown to be highly enriched so that they may constitute potential relevant biomarkers (Akers et al., 2013; Yang et al., 2017).

Taking into account the crosstalk between GAMs in glioma, it is also important to highlight that recent studies showed that microglia-derived exosomes in the brain could be used as nanotherapeutic agents against glioma cells (Murgoci et al., 2018). In fact, extracellular ATP promotes microglia activation and induces the release of EVs, which can potentially mediate intercellular communication between microglia and the microenvironment (Drago et al., 2017). Furthermore, it was also demonstrated that the crosstalk between GB and astrocytes occurs through EVs. GB EV-treated NHAs had increased the expression of IFN-γ, ILs 12, 1A, 8, and 1B, chemokine CXCL10, and factor C5. Moreover, the conditioned medium of GB EV-treated NHAs induces the growth of GB cells. Thus, miR-9 released from Tat-stimulated astrocytes can be taken up by microglial cells, which result in their migratory phenotype. Therefore, glial crosstalk via miRNAs released from EVs play a crucial role mediating pathogenesis, which could eventually lead to the development of novel therapeutic strategies aimed at cushion neuropathogenesis (Yang et al., 2017).

## How to Manipulate Microglia/Astrocytes–Glioblastoma Crosstalk

There are many efforts to overcome the GB’s resistance by finding and testing new compounds that can be used as therapeutic approaches. One of the main targets to these compounds are the neighbor entities of GBs, such as the microglia and astrocytes, since they have a great influence on the tumor progression (Hambardzumyan et al., 2016; Matias et al., 2018; Roesch et al., 2018).

We will present the most recent works that demonstrate to be potential therapeutic approaches to target microglia and astrocytes, which could turn off the immunosuppression observed in GBs and decrease their progression.

According to the literature, the tumor progression is facilitated by the immunosuppressive microenvironment created by GB and GAMS crosstalk (Hambardzumyan et al., 2016). In this regard, targeting microglial cells may be useful in manipulating GB growth, especially the GSCs (Ye et al., 2012; da Fonseca et al., 2016). Possibly, the therapeutic approach that can be used by chemoattractant receptors/ligands blockage to reduce GAMs recruitment, or even depleting or re-polarizing to enrich the pro-inflammatory M1-like GAMs, is still under discussion (Poon et al., 2017).

Among the therapeutic drugs targeting microglia in a preclinical model, the blockage of CSF-1R signaling in glioma-bearing mice, through the anti-CSF-1R antibody Pexidartinib (PLX3397), resulted in a significant reduction tumor infiltration of GAMs and, consequently, a decrease in tumor volume, as well as an apparent increase of the survival time of treated mice (Pyonteck et al., 2013). However, later on, Butowski et al. (2016) showed that PLX3397 was well tolerated in patients and readily crossed the blood–tumor barrier but showed no efficacy.

Also, the CSF-R1 inhibitor BLZ945, in glioma-bearing mice, demonstrated reduced expression of M2-like markers in GAMs, which – after the administration of the insulin-like growth factor 1 (IGF-1) and phosphatidylinositol-4,5-bisphosphate 3-kinase (PI3K) antagonist – reduced the tumor progression, and an increase in the survival time of mice was observed (Coniglio et al., 2012). In agreement with that, Quail et al. (2016) concluded that the dual inhibition of IGF-1R/PI3K and CSF-1R significantly prolonged overall survival rate in recurrent glioma in mice.

The chemokines and their receptors are highly expressed in microglial cells, and they are extremely important to tumor progression (Zhao et al., 2015; Laudati et al., 2017; Jin et al., 2018). Thus, they may constitute a good target to decrease the microglia and tumor interaction. For that reason, many studies have been made to discover new inhibitors of chemokines receptors. Recently, it has been shown that the CCR5 blocker MRV was able to reduce the M2-like phenotype by reducing the ARG-1 and IL-10 proteins and promoting the upregulation of M1-like markers, such as NO and IL-1β in microglial cells treated in GB-conditioned medium (Laudati et al., 2017). The inhibition of the SDF-1 receptor (CXCR4) by using AMD3100 is another approach to target GAMs. Also, the inhibition of SDF in myeloid cells was performed using the Plerixafor in multiple myeloma and lymphoma, which constitutes a huge promise regarding glioma (Dillmann et al., 2009; Rios et al., 2016).

In the search to find an efficient anti-angiogenic therapy to treat GB, it was recently demonstrated that the survival of GB-xenografts mice on the isolated anti-VEGF blockade was derived from the double blockade of Ang-2/increased VEGF. This dual blockade induced the reprogramming of GAMs from the pro-tumor M2-like phenotype toward the antitumor M1-like phenotype (Kloepper et al., 2016).

Moreover, another antagonist of CXCR4, the peptide R, decreases the tumor mass of GB-xenotransplanted cells and reduces the CD11/CD68-positive cells in peritumoral zone of the tumor. In addition, the peptide R induces the M1-like phenotype in microglial cells by increasing the iNOS levels (Mercurio et al., 2016).

In parallel, AXL is highly expressed by GBs and microglia secrete the protein S (PROS1) responsible for phosphorylating AXL, which consequently induces the aggressiveness of GBs. Thus, Sadahiro et al. (2018) verified if the combination of a specific inhibitor of AXL (BGB324) with Nivolumab (antibody against PD-1) could be used for GB treatment. They showed increased survival in mice treated with BGB324 and Nivolumab. Further, in this study, the authors observed that protein S (PROS1)/tyrosine-protein kinase (AXL) pathway is involved in extrinsic immune microenvironment by reduction of microglia infiltration after treatment (Sadahiro et al., 2018).

Moreover, *in vitro* and *in vivo* studies showed that the high expression of Nrp1 is correlated with the poor prognosis by inducing the proliferation and migration of GB cells. It has been shown that the Nrp1 is important to induce M2-like phenotype in microglia. Studies showed that the specific inhibitor of Nrp1 named EG00229 reduced tumor growth. These findings suggest that targeting Nrp1 could be a useful therapeutic approach for GBs since the EG00229 reduces Nrp1 expression and induces the M1 polarized phenotype on microglial cells, which consequently suppress the vascularization and proliferation of tumor cells (Miyauchi et al., 2016).

The phenolic compound, chlorogenic acid (CHA), has an anti-tumor effect in multiple malignant tumors, including GB. *In vitro* studies showed that CHA inhibited GB growth and induced apoptosis of GB cells. CHA promotes M1 polarization by increasing the levels of iNOS, MHC II, and CD11c and, consequently, reduces the M2 polarized markers by decreasing ARG and CD206 levels. Besides, the CHA reduced the tumor size and increased the number of CD11c^+^ M1 GAMs compared to CD206^+^ M2 GAMs in tumor tissue from mice orthotropic xenograft models of GB (Xue et al., 2017).

Histopathological studies demonstrated an increase of infiltrated microglia in chondroitin sulfate proteoglycan 4 (CSPG4), which is highly expressed in GB samples, co-expressing TNF-α. Recently, regarding the new era of immunotherapy, Pellegatta et al. (2018) showed that CSPG4 can be targeted by CAR-T cells, promoting the control of tumor growth by inducing TNF-α expressed from microglial cells, leading to tumor cells death.

Together with microglial cells, astrocytes are also a crucial part of the TME cells that are involved in similar interactions with the tumor cells influencing its malignancy.

In this sense, it is known that hypoxic microenvironment maintains the malignant profile of GBs through the HIF-1α expression. However, in a hypoxic microenvironment, there are still astrocytes, which can release high levels of CCL20 that, upon binding to the GB receptor CCR6, induce the expression of HIF-1α (Jin et al., 2018). The authors noticed an increase in the tumor mass that was accompanied by a high vascularization and increased levels of HIF-1α in mice injected with GB cells compared to CCR6-knockdown GB xenografts. These results suggest that, in a hypoxic microenvironment, GB cells growth is dependent on the CCL20 derived from astrocytes. Thus, the CCL20/CCR6 signaling could be a potential therapeutic target for GB treatment (Jin et al., 2018).

Some evidence support the idea that astrocytes can influence the tumor chemoresistance by protecting GB cells to TMZ or Vincristine (VCR) cytotoxicity by increasing the CX43. Therefore, it can be considered a potential target for the CX43-mediated GJCs between GB/astrocytic cells, and so the combination of the CX43 inhibitor with TMZ or VCR could be a successful therapy to GBs (Chen et al., 2015).

On the other hand, Hong et al. (2015) showed that the gap junctions play a role during glioma and astrocyte interactions by allowing the transference of miRs from glioma cells to astrocytes. So, the miR-4519 and miR-5096-transferred from GB to astrocytes through CX43 induce the pro-invasive phenotype in astrocytes (Hong et al., 2015). This evidence supports the idea that CXs and miRs may be a useful therapeutic target to decrease the interaction between astrocyte and GB and, consequently, decrease tumor progression.

As previously mentioned, the EVs are used by tumor cells as carriers to communicate with other cells from TME, such as astrocytes (Oushy et al., 2018). GB EVs modulate NHAs by increasing their migratory capability and inducing the cytokine production (as ILs 4, 10, and CCL2), which consequently stimulate the tumor growth (Oushy et al., 2018). Therefore, finding a way to control the release of these GB EVs may be one possible strategy to decrease the tumor progression.

The principal studies previously mentioned regarding microglia/astrocytes GB crosstalk-therapeutic targets are summarized in **Table 2**.

**Table 2:** Microglial/astrocytic cells – therapeutic targets in GB.

Therapy	Therapeutic target	Target cells	Study phase	References
Pexidartinib	CSF-1R blockade	Microglia	Phase I clinical trials	Butowski et al., 2016
BLZ945	CSF-1R inhibition combined with IGF-1R and PI3K blockade	Microglia	Preclinical studies	Coniglio et al., 2012; Quail et al., 2016
Maraviroc (MRV)	CCR5 blockade	Microglia	Preclinical studies	Laudati et al., 2017
AMD3100	SDF-1 receptor (CXCR4) inhibition	Microglia	Preclinical studies	Dillmann et al., 2009
Peptide R	SDF-1 receptor (CXCR4) inhibition	Microglia	Preclinical studies	Mercurio et al., 2016
BGB324 and Nivolumab	AXL and PD-1 inhibition	Microglia	Preclinical studies	Sadahiro et al., 2018
EG00229	Nrp1 blockade	Microglia	Preclinical studies	Miyauchi et al., 2016
CART cells	CSPG4 inhibition	Microglia	Preclinical studies	Pellegatta et al., 2018
Chlorogenic acid (CHA)	Arg and CD206	Microglia	Preclinical studies	Xue et al., 2017
CCL20	CCL20/CCR6 signaling inhibition	Astrocytes	Preclinical studies	Jin et al., 2018
CX43 inhibitor with TMZ or VCR	CX43	Astrocytes	Preclinical studies	Chen et al., 2015

## Conclusion and Future Perspectives

During recent years, many efforts have been made to understand the heterogeneity of microglia/astrocytes. However, still little is known about the complex interaction and dynamics among microglia/astrocytes and tumor, such as GB, and how this crosstalk can influence tumor growth and patient’s outcome. Nevertheless, to successfully improve GB patient survival, more in-depth knowledge is needed regarding the crosstalk between those TME cells.

In this review, we discussed the several factors and proteins already known that can modulate the activity of microglia or astrocytes during GB progression. Cytokines, chemokines, neurotrophic, and morphogenic factors can be associated with recruitment of tumor parenchyma cells, such as microglia and astrocytes, by the modulation of their phenotypes in order to influence tumor growth. In parallel, metabolic changes are known to be one of the more important engines for cell mechanisms, which include the cell proliferation and migration. In this regard, it is necessary to clarify the role of metabolites and enzymes of the cycle Krebs, as well as the glycolysis components during microglia/astrocytes–GB crosstalk. Also, the modulation of the cytoskeleton and ECM proteins is crucial for this crosstalk. However, more studies are needed to understand the underlying morphological changes during M1 and M2 polarization in microglia and astrocyte reactivity.

On the other hand, the miRNAs have been described as positive or negative regulators of this crosstalk, such as miR451/miR21 that are involved in microglia polarization into M1/M2-like phenotype. Thus, the identification of new miRNAs that can regulate this interaction is one of the main focus for future investigations.

The communication between microglial/astrocytes and GBs cells can be performed through the EVs. In fact, EVs can participate in this interaction through the transport of several factors, such as miRNAs or even morphogenic factors, such as the Shh or Wnt. Also, the ABC transporters have a huge importance in GB resistance. Additionally, these transporters may also be involved in the transference of factors that modulate microglia/astrocytes behavior. Thus, it seems to us that the development of novel therapies, targeting factors/receptors that modulate the phenotype of microglial and astrocyte cells toward a proinflammatory behavior, may be a useful approach for GB patients. **Table 2** represents some studies that are in clinical stages, and their results, such as PLX3397, have not been very promising. However, some studies have demonstrated the efficacy of compounds targeting membrane receptors on microglial cells, such as Plerixafor, peptide R, and the combination of BGB324 with Nivolumab. Using the natural ability of the EVs as transporters, it may be possible to use them as drug delivery strategies, thus targeting the microglial and astrocytic cells.

In a clinical setting, it seems to be crucial to identify new potential targets in the microglia/astrocytes–GB crosstalk and, ultimately, develop a new range of therapeutic approaches, hopefully contributing in future to a better outcome for patients with an aggressive and deadly tumor, such as GB.

## Author Contributions

The review was conceptualized, written, edited, and critically evaluated by each of the authors. DM and JB-S had equal contribution for the literature search and article final preparation. The work was supervised by VF, TS, and VM-N. All authors read and approved the final submitted version of the manuscript.

## Conflict of Interest Statement

The authors declare that the research was conducted in the absence of any commercial or financial relationships that could be construed as a potential conflict of interest.
